# Mr. Madden's Travels in Turkey, Egypt, Nubia, and Palestine

**Published:** 1829-07-01

**Authors:** 


TRAVELS IN TURKEY, EGYPT, NUBIA, AND PALESTINE, IN 1824-5-6 AND 7. By *R. R. Madden*, Member of the Royal College of Surgeons. Two vols. 8vo. June, 1829.

THERE is no profession, or even trade, so easily carried from country to country, and at the same time so marketable, as PHYSIC. The lawyer requires a cart-load of statutes and precedents—and after all they would be useless, the moment he leaves his native land. The divine indeed may carry a good stock of theology in his head, yet he finds it a very unsaleable commodity, except among savages. But, as men die in every country, and generally fall ill before they die, so the doctor is every where sure of employment—even among the rankest fatalists, or professors of Islamism. The principles of our *science* are of course, carried in our heads:—and the instruments of our *art*—the *materiel* with which we work, may often be enfolded in our turbans, when in Turkish costume. A sharp lancet—an ounce or two of calomel—a few packets of James's powder—and some sulphate of quinine, will generally prove the essential articles. Most of the auxiliaries in the *methodus medendi* may be found indigenous in the countries through which we travel. This being the case, we think we may, without prejudice or partiality, affirm that a medical man, *ceteris paribus*, is the best calculated for travelling through foreign realms, especially through those where science and civilisation are still in the rear. The medical character is far more sacred than the clerical in such countries. Physic interferes not with men's moral, political, or religious opinions. It removes, or professes to remove bodily ailments, leaving the patient to his own doctrines and inclinations in all other respects. It was well observed by the Earl of Blessington, to the author of these travels, previous to his embarkation at Naples, that—" the medical profession afforded the best passport to a traveller in the East; and gave him access to the habitations, and even to the HAREMS of a people, whose prejudices debarred other Europeans from that intercourse with them, which is essential to a knowledge of their modes and customs." The author soon found the truth of these observations; but, as Herodotus was restrained by religious awe, from disclosing the secrets of the sanctuary, when treating of the worship of the Egyptians, so our judicious traveller allows himself only to describe, in the *penetralia* of the harem, what is fit to reach the ear—perhaps a little less than what met the eye.

Mr. Madden appears to possess, independent of his professional character, many essential qualifications of a traveller. These are, youth, strong physical constitution, zeal almost romantic, a temper peculiarly placid, (which, by the way, contributed much to the success of his enterprizes, and, in many instances, saved his life)—facility of acquiring languages, good general education, great readiness and even happiness in clothing his ideas in easy, not to say elegant, dress. We are exceedingly glad that he either possessed not, or exercised not, a mania for exploring—

"Antiquities of nations now no more."

Instead of employing his time in measuring the altitudes and dimensions of every old pillar, temple, or pyramid, which fell in his way, together with all this kind of research, Mr. Madden travelled with the eye of a painter, and the mind of a philosopher—drawing accurate portraits of men and things, as they are—and reflecting, in his leisure moments, on what he had seen and described. The result has been one of the most amusing and instructive books of travels which we have ever perused. Were this a general not a medical review, we should proceed to prove the truth of what we have advanced; but the nature of this Journal limits us to the notice only of some of the medical remarks and facts contained in the volumes before us. Considering that the work is destined for general readers, the medical portions of it cannot be subjected to rigid professional criticism, since it is obvious that the author could not put them in a shape or condition calculated for exclusive medical perusal. Nevertheless, they will be found by no means undeserving of notice.

*Practice of Physic in Constantinople.* There are about 50 medical practitioners in the renowned city of Constantine, principally Franks from Italy and Malta—a few Ionian Greeks, and Armenians. There are two regularly educated English physicians, highly respected by the disciples of Cross and Crescent. The following passage may appear very droll—but it is not so very different from the customs of cities farther North than Constantinople, as some may imagine.

"Every *medico* has his allotted quarter; he beats this ground daily in pursuit of patients, and visits all the coffeehouses in the district with a Greek *drogueman*, as interpreter, at his heels, whose occupation it is to scent out sickness, and to extol the doctor. They are ever to be found on the most public bench of the coffeeshop, smoking with profound gravity, and prying into the features of those around them, for a symptom of disease. I confess I had to descend to this degradation, to get practice, in order to become acquainted with the domestic customs of the people. The first day my drogueman, who had just left the service of a Roman doctor, and had been practising on his own account since his discharge (for all droguemen become doctors), took upon him to teach me my professional duty, which he made to consist, in never giving advice before I got my fee, in never asking questions of the sick, and in never giving intelligible answers to the friends; I was to look for symptoms only in the pulse; I was to limit my *prognosis* to three words, '*In Shallah*,' or, 'Please the Lord,' for doubtful cases; and '*Allakharim*,' or, 'God is great,' for desperate ones. I took my post in the coffeeshop, had my pipe and coffee, while my drogueman entered into conversation with the Turks about us. I soon heard him narrating a history of a miraculous cure which he had seen me perform some days before, on the body of a dying Effendi; how I had taken out his liver and put it in again, after scraping off the disease, and how the patient got well the next day, and gave me five purses. I was exceedingly annoyed; but the fellow seemed to mind my anger little, and even reproved 'my want of prudence' with a frown." 56.

The Turks swallowed this story, and Mr. Madden got two patients before he left the coffee-house—the fee from one being a cup of coffee—that of the other, 50 piastres. For the latter fee, which was a good one, he had to undertake the cure of an old and ugly woman, respecting whose case he could only get a touch of the pulse through a partially open door, for the whole of his information. Our author's practice, however, increased more rapidly than a doctor's practice usually does in more civilized capitals. His description of a great medical consultation on the case of a Turkish grandee, in which a priest took the lead and decided on the remedy, would be laughable in the extreme, were it not accompanied by the reflexion that the oil of wax, which was prescribed, contained, in all probability, a fatal dose of poison! The bulk of the patient's property was invested in a mosque. Mr. Madden having reason to suspect the probity of the priest, gave a hint to one of the attendants, that the grandee would die if he took the medicine; but it is probable the hint was not taken, as the grandee soon expired.

Mr. Madden's description of the opium-eaters is very spirited, as indeed are all his descriptions. We must make room for a short extract.

"I had heard so many contradictory reports of the sensations produced by this drug, that I resolved to know the truth, and, accordingly took my seat in the coffeehouse, with half a dozen *Theriakis*. Their gestures were frightful; those who were completely under the influence of the opium talked incoherently, their features were flushed, their eyes had an unnatural brilliancy, and the general expression of their countenances was horribly wild. The effect is usually produced in two hours, and lasts four or five: the dose varies from three grains to a drachm. I saw one old man take four pills, of six grains each, in the course of two hours; I was told he had been using opium for five and twenty years; but this is a very rare example of an opium eater passing thirty years of age, if he commence the practice early. The debility, both moral and physical, attendant on its excitement, is terrible; the appetite is soon destroyed, every fibre in the body trembles, the nerves of the neck become affected, and the muscles get rigid; several of these I have seen, in this place, at various times, who had wry necks and contracted fingers; but still they cannot abandon the custom: they are miserable till the hour arrives for taking their daily dose; and when its delightful influence begins, they are all fire and animation. Some of them compose excellent verses, and others address the bystanders in the most eloquent discourses, imagining themselves to be emperors, and to have all the harems in the world at their command." 25.

Our inquisitive traveller tried the experiment on himself, and after taking four grains of opium in divided doses, began to think himself among the HOURIS of Paradise.

"My faculties appeared enlarged: every thing I looked at seemed increased in volume; I had no longer the same pleasure when I closed my eyes which I had when they were open; it appeared to me as if it was only external objects, which were acted on by the imagination, and magnified into images of pleasure: in short, it was 'the faint exquisite music of a dream' in a waking moment. I made my way home as fast as possible, dreading, at every step, that I should commit some extravagance. In walking, I was hardly sensible of my feet touching the ground, it seemed as if I slid along the street, impelled by some invisible agent, and that my blood was composed of some etherial fluid, which rendered my body lighter than air. I got to bed the moment I reached home. The most extraordinary visions of delight filled my brain all night. In the morning I rose, pale and dispirited; my head ached; my body was so debilitated that I was obliged to remain on the sofa all the day, dearly paying for my first essay at opium eating." 27.

Mr. Madden has given a short and animated description of the climate of Egypt. About the end of June, a heavy dew, called the nocta, begins to fall—the drooping plants again revive—the plague ceases—and a new disease is ushered in, dysentery—which is of a very severe kind.

"I was seized with dysentery, in consequence of leaving my window open at night, the first week of the Nocta. An Italian physician, of the greatest eminence, who had failed as a merchant, was brought to me by my friends. I did not wish it, but they desired it, as my danger was imminent. The first thing he proposed, was to put ice to my abdomen, and ordered me to drink nothing but ice, '*to keep down the inflammation.*' I thought this very strange; I objected to it, but he silenced me by the assurance that he had practised fifteen years in the country, and had found ice the only specific for dysentery. My mind was enfeebled by the disease, as well as my body; I suffered him to apply the ice: two hours after it, my symptoms were fearfully augmented; the pain was excruciating; and I thought my last tour on earth was nearly finished, and that my mortal pilgrimage was to end in Alexandria.

"I gave orders to admit the doctor no more; I took scruple doses of calomel, for three successive days, twice a day; the third day my mouth was affected, and from that moment every bad symptom ceased. Did this quondam merchant want to dispatch me, or was his remedy prescribed through mere ignorance? I dare not determine." 220.

Mr. Madden went to the East a decided disciple of Dr. Maclean—a stanch non-contagionist, as far as plague is concerned. At Constantinople he had the treatment of 46 cases. In his opinions there he was constantly perplexed—one day supposing the disease contagious, another day that it was infectious, and a third that it was neither. One thing only was constant and unequivocal—the fatal termination! "Almost every patient I treated or saw treated in Constantinople, was bled, purged, and vomited, to keep down the *inflammatory fever*—and every patient died exhausted."

"It was only in Candia I had reason to alter my opinion about Dr. M'Clean's doctrines. I was there thoroughly persuaded that plague is both *contagious and infectious, at one period epidemical, at another endemical.* In plain English, that the miasma may be communicated by the touch, or by the breath; that at one period it is confined to a particular district, and at another is disseminated amongst the people. But if plague have one form more decided than another, it is the endemic. I survived the folly of inhaling the infected atmosphere of plague chambers, sitting on the bedside of pestilential people, as I was in the habit of doing at Constantinople, and feeling their ill conditioned sores. In Candia, I say 'the fell serjeant' was too often at my elbow to let me sleep in the sweet security of non-contagion. The effluvia of pestilence has been too near my nostrils to continue to court approximation, for the sake of supporting a system." 264.

"The disease which plague most resembles is the gaol fever of this country, bad typhus fever; and in contradistinction to *typhus gravior*, or putrid fever, I have given plague the name of *typhus gravissimus.* The symptoms from the first are general debility, congestion about the heart, not depending on inflammation, but on the putrescent state of the circulation. It differs little from putrid typhus, except in its duration and eruptions. In every stage of plague, nature appears to lie prostrate under the influence of the poisonous miasma; and when the patient sinks at last, it is from the want of force in the constitution to drive out the eruptions on the surface." 265.

We shall reserve the article on Plague, however, for our next number, when we shall present our readers with several other medical morceaus from these highly interesting volumes.

